# Genome-wide microRNA profiling of human temporal lobe epilepsy identifies modulators of the immune response

**DOI:** 10.1007/s00018-012-0992-7

**Published:** 2012-04-26

**Authors:** Anne A. Kan, Susan van Erp, Alwin A. H. A. Derijck, Marina de Wit, Ellen V. S. Hessel, Eoghan O’Duibhir, Wilco de Jager, Peter C. Van Rijen, Peter H. Gosselaar, Pierre N. E. de Graan, R. Jeroen Pasterkamp

**Affiliations:** 1 Department of Neuroscience and Pharmacology, University Medical Center Utrecht, Universiteitsweg 100, 3584 CG, Utrecht, The Netherlands; 2 Department of Pediatric Immunology, University Medical Center Utrecht, Universiteitsweg 100, 3584 CG, Utrecht, The Netherlands; 3 Department of Neurology and Neurosurgery, University Medical Center Utrecht, Universiteitsweg 100, 3584 CG, Utrecht, The Netherlands

**Keywords:** Mesial temporal lobe epilepsy, Nuclear localization, Immune regulation, Immune system, MicroRNA, RNA profiling, Temporal lobe epilepsy

## Abstract

Mesial temporal lobe epilepsy (mTLE) is a chronic neurological disorder characterized by recurrent seizures. The pathogenic mechanisms underlying mTLE may involve defects in the post-transcriptional regulation of gene expression. MicroRNAs (miRNAs) are non-coding RNAs that control the expression of genes at the post-transcriptional level. Here, we performed a genome-wide miRNA profiling study to examine whether miRNA-mediated mechanisms are affected in human mTLE. miRNA profiles of the hippocampus of autopsy control patients and two mTLE patient groups were compared. This revealed segregated miRNA signatures for the three different patient groups and 165 miRNAs with up- or down-regulated expression in mTLE. miRNA in situ hybridization detected cell type-specific changes in miRNA expression and an abnormal nuclear localization of select miRNAs in neurons and glial cells of mTLE patients. Of several cellular processes implicated in mTLE, the immune response was most prominently targeted by deregulated miRNAs. Enhanced expression of inflammatory mediators was paralleled by a reduction in miRNAs that were found to target the 3′-untranslated regions of these genes in reporter assays. miR-221 and miR-222 were shown to regulate endogenous ICAM1 expression and were selectively co-expressed with ICAM1 in astrocytes in mTLE patients. Our findings suggest that miRNA changes in mTLE affect the expression of immunomodulatory proteins thereby further facilitating the immune response. This mechanism may have broad implications given the central role of astrocytes and the immune system in human neurological disease. Overall, this work extends the current concepts of human mTLE pathogenesis to the level of miRNA-mediated gene regulation.

**Electronic supplementary material:**

The online version of this article (doi:10.1007/s00018-012-0992-7) contains supplementary material, which is available to authorized users.

## Introduction

Temporal lobe epilepsy (TLE) is a neurological condition characterized by recurrent seizures that originate from the temporal lobe. TLE accounts for one-third of all patients with epilepsy [1] and can be divided into several subgroups including mesial TLE (mTLE). mTLE is associated with characteristic pathological features and about 30 % of mTLE patients are pharmaco-resistant [2]. The pathological mechanisms underlying mTLE are poorly understood. Recent studies show that patterns of gene expression are significantly altered in experimental and human mTLE [3–7]. Thus, the regulatory mechanisms that normally control gene expression may be affected. For example, it was recently shown that transcriptional repressors play a key role in acquired HCN1 channelopathy [8]. Insight into how regulation of gene expression is altered may provide important new insights into mTLE pathogenesis and could yield novel therapeutic targets.

During the past several years, microRNAs (miRNAs) have emerged as important post-transcriptional regulators of gene expression, providing a completely new level of control of gene expression. miRNAs are short (20–23 nucleotides), non-coding RNAs that recognize partially complementary target sequences in select mRNAs and predominantly inhibit protein expression by either destabilizing their mRNA targets or by inhibiting protein translation [9–13]. miRNA-mediated mechanisms have been shown to contribute to the regulation of brain development and homeostasis [11, 14] and can be affected in neural diseases such as multiple sclerosis [15–17].

Recent animal studies support the hypothesis that miRNAs may contribute to the pathogenesis of epilepsy [18–21]. To systematically assess the role of miRNAs in human mTLE, we conducted the first genome-wide miRNA expression profiling study of human mTLE patients. The observed microRNA signatures led us to (1) unveil a marked aberrant expression and nuclear localization for miRNAs in mTLE patients, and (2) identify components of the immune response as targets of the most strongly regulated miRNAs in mTLE.

## Materials and methods

### Patient selection and tissue collection

Hippocampal tissue samples of pharmaco-resistant mTLE patients were obtained after surgery at the University Medical Centre Utrecht. Patients were selected for surgery according to the criteria of the Dutch Epilepsy Surgery Program [22]. The excision was based on clinical evaluations, interictal and ictal EEG studies, MRI and intraoperative electrocorticography. Informed consent was obtained from all patients for all procedures approved by the Institutional Review Board. Immediately after en bloc resection of the hippocampus, the tissue was cooled in physiological saline (4 °C) and cut on a precooled plate into three slices perpendicular to its longitudinal axis. The two outer parts were used for pathological analysis. In the operating room, the middle section was divided into a part that was immediately frozen on powdered dry ice and a part that was immersion-fixed in 4 % paraformaldehyde in 0.1 M phosphate buffer for 24 h at 4 °C. Following fixation, tissue was embedded in paraffin and stored at 4 °C. Frozen samples were stored at −80 °C. Frozen and paraffin-embedded control hippocampal tissue samples were obtained from postmortem cases without hippocampal aberrations from the Netherlands Brain Bank (www.brainbank.nl). All control material was collected from donors with written informed consent for brain autopsy and the use of the material and clinical information for research purposes. Prior to dissection, brain pH was measured interventricularly using an 18-GA 3.50-in. spinal needle. Detailed histological examination of the hippocampal material from all patients used in this study showed that all samples were devoid of tumor tissue. All patient samples used in this study had RIN values >6 (range 6.4–8.4; mean 7.2) [23–25] confirming that all RNA samples were of excellent quality. Table 1 provides a summary of the clinical data of all patients included in the study. Hippocampal specimens were divided into three groups: a non-epileptic autopsy control group (control, *n* = 10), a group of mTLE patients without signs of hippocampal sclerosis (mTLE−HS, *n* = 10) and an mTLE group with hippocampal sclerosis (mTLE + HS, *n* = 10). The severity of HS was determined by an experienced neuropathologist using the Wyler classification method [26] defining W0 as hippocampal tissue without HS and W4 as tissue with the most severe type of HS. Wyler classification was independently verified on paraffin-embedded tissue.

**Table 1:** Clinical data of mTLE and autopsy control patients

Patient group	Age (years)	Gender	Age of onset (years)	COD/pathology	PMD (h)	Brain pH	RIN values	AED’s	Engel score
1) Control	50	F	–	Metastasized broncocarcinoma	4	6.98	6.9	–	–
2) Control	58	M	–	Unknown. ALS patient	7	6.46	6.4	–	–
3) Control	62	M	–	Unknown, non-demented control	7	6.36	6.4	–	–
4) Control	73	F	–	Subdural hematoma	6.5	n.d.	8.4	–	–
5) Control	71	M	–	Pancreas carcinoma	9	6.64	7.5	–	–
6) Control	64	F	–	Respiratory failure	4.5	6.45	8	–	–
7) Control	70	M	–	Sepsis with broncopneumonia	20.5	6.68	6.5	–	–
8) Control	94	F	–	CVA	4	6.68	6.8	–	–
9) Control	48	M	–	DMT I-induced organ failure	5.5	6.88	7.8	–	–
10) Control	74	M	–	Pulmonary carcinoma	8	6.87	7.9	–	–
Median	67	M6, F4	–	–	6.75	6.68	7.2	–	–
11) TLE−HS	45	M	18	W0, FCD type1 to 2A in cortex	–	–	8.1	LTG, PHT	I A
12) TLE−HS	46	F	16	W0, MCD type 1 in cortex	–	–	8.2	CBZ, VPA	I A
13) TLE−HS	46	M	33	W0, epilepsy after head trauma	–	–	7.1	CBZ, VPA, TPR	I A
14) TLE−HS	42	F	20	W0, DNT WHO grade I	–	–	8.3	CBZ, LTG, LEV	I A
15) TLE−HS	34	F	24	W0, cortical cavernoma	–	–	7.4	CBZ	I A
16) TLE−HS	40	F	17	W0, MCD type 1 in cortex	–	–	8	LEV, LTG, CBZ	I A
17) TLE−HS	43	F	10	W0, therapy-resistant epilepsy	–	–	8.1	PHT, LTG	I A
18) TLE−HS	47	M	16	W0, therapy-resistant epilepsy	–	–	8.6	CBZ, VPA, LTG, LEV	II A
19) TLE−HS	28	M	12	W0, therapy-resistant epilepsy	–	–	7.4	CBZ, TPR.	I A
20) TLE−HS	54	M	35	W0, ganglioglioma WHO grade I	–	–	8.2	OXC, LTG, CLO	I A
Median	44	M5, F5	17.5	–	–	–	8.1	–	–
21) TLE + HS	49	F	12	MTS W4	–	–	8	OXC, CLO	I A
22) TLE + HS	44	F	13	MTS W2	–	–	7.9	CBZ, OXC, CLO	I A
23) TLE + HS	41	M	1	MTS W4	–	–	8.1	CBZ	I A
24) TLE + HS	52	F	20	MTS W4	–	–	8.1	CBZ, CLO, DZP	I A
25) TLE + HS	50	M	2.5	MTS W4	–	–	8.6	CBZ, GBP	II A
26) TLE + HS	36	F	14	MTS W4	–	–	7.6	OXC, LZP	n.d.
27) TLE + HS	42	M	0.7	MTS W4	–	–	8.1	LEV, LTG	II A
28) TLE + HS	36	M	10	MTS W4	–	–	8.2	OXC, PGB	I A
29) TLE + HS	41	M	0.6	MTS W4	–	–	9.2	PHT, CLO, CBZ, LTG	I A
30) TLE + HS	42	F	8	MTS W4	–	–	9.1	LEV, LTG, PBT	I A
Median	42	M5, F5	9	–	–	–	8.1	–	–

*COD* cause of death, *PMD* postmortem delay, *RIN* RNA integrity number, *AED* anti-epileptic drug, *n.a.* not applicable, *CVA* cerebrovascular accident, *ALS* amyotrophic lateral sclerosis, *DMT I* diabetes mellitus type I, *W0*–*W4* Wyler score, *FCD* focal cortical dysplasia, *WHO grade* world health organization grading scale of malignancy, *LTG* lamotrigine, *PHT* phenytoin, *CBZ* carbamazepine, *VPA* valproinic acid, *TPR* topiramate, *LEV* levetiracetam, *OXC* oxcarbazepine, *CLO* clobazam, *DZP* diazepam, *GBP* gabapentin, *LZP* lorazepam, *PGB* pregabaline, *PBT* phenobarbital

### RNA isolation and quality control

Nissl stained cryo-sections were generated to ensure that all anatomical regions were equally represented in each sample. For the purpose of RNA isolation, 25-μm-thick cryo-sections were cut until approximately 20 mg of tissue was collected. This material was stored at −80 °C until all samples were collected. Then, all samples were thawed and processed in parallel in QIAzol lysis reagent to prevent RNA degradation. Total RNA was extracted using the miRNeasy kit (Qiagen) according to the manufacturer’s protocol. RNA quality (RIN value; Table 1) was assessed using a RNA 6000 Nano chip on the 2100 Bioanalyzer (Agilent) and RNA quantity was determined using Nanodrop (Thermo Scientific).

### microRNA array

1,000 ng total RNA per patient sample and reference (pool of all samples) was labeled with Hy3™ and Hy5™ fluorescent labels, respectively, using the miRCURY™ LNA Array power labeling kit (Exiqon) following procedures described by the manufacturer. Hy3™-labeled samples and a Hy5™-labeled reference RNA sample were mixed pair-wise and hybridized to the miRCURY™ LNA Array version 5th Generation (Exiqon), which contains capture probes targeting all human (hsa) miRNAs registered in miRBASE version 15.0 at the Sanger Institute. The hybridization was performed according to the miRCURY™ LNA array manual using a Tecan HS4800 hybridization station (Tecan). After hybridization, microarray slides were scanned and stored in an ozone free environment (ozone level below 2.0 ppb) in order to prevent bleaching of the fluorescent dyes. The miRCURY™ LNA array microarray slides were scanned using the Agilent G2565BA Microarray Scanner System (Agilent) and image analysis was carried out using ImaGene 8.0 software (BioDiscovery). The quantified signals were background corrected (Normexp with offset value 10) [27] and normalized using the global Lowess (LOcally WEighted Scatterplot Smoothing) regression algorithm.

The 130 miRNAs that passed the filtering criteria on variation across samples were submitted to a principal component analysis (SD > 0.50) to identify general similarities and differences. Additionally, a univariate general linear model analysis was applied with age and gender as covariates using the top 30 changed miRNAs. This analysis did not result in loss of Bonferroni corrected significance for any of the groups (see Online Resource 2c) and nor did it result in significant *p* values for either age or gender (*p* > 0.05). As pH and PMD are potential confounders only in autopsy control patients, we performed additional Pearson’s correlations tests on the top 30 changed miRNAs against pH and PMD in this group. No significant correlations were detected for pH and PMD.

### Quantitative PCR

Based on the array profiling data, miR-29a and miR-423-3p were identified as normalization miRNAs using NormFinder [28] showing minimal variation across all samples (SD = 0.17). Total RNA from all control or nine mTLE + HS (Wyler 4, excluding patient #22) patients was pooled and used for first strand cDNA synthesis using a universal cDNA synthesis kit (Exiqon). Duplicate cDNAs were generated for each RNA pool. Quantitative PCR reactions were run on a 7900HT Real-Time PCR System (Applied Biosystems) using microRNA LNA™ PCR primer sets and SYBR Green master mix (Exiqon). All samples were run in duplicate and the base for exponential amplification was verified to be two by standard curve analysis for all primer sets. *C*_t_ values were determined using SDS software (Applied Biosystems) with manual baseline and threshold settings. Normalization and expression analysis based on the DC_t_ method was performed using Qbase version 1.3.5.

### In situ hybridization

Non-radioactive in situ hybridization was performed on slides that contained three sections, one of each patient group. Per group, six of the ten patients used for the microarray study were analyzed. In situ hybridization and subsequent immunohistochemistry were performed on 7-μm-thick paraffin sections as described previously [29]. Briefly, sections were deparaffinated, acetylated (10 min at RT) and treated with proteinase K (5 μg/ml 5 min at RT). Prehybridization was performed for 1 h at RT. Hybridization was performed with 10–20 nM double-DIG (3′ and 5′)-labeled locked nucleic acid (LNA) probe for human miR-124, miR-20a, miR-92b, miR-193a-3p, miR-138, miR-221, miR-222, miR-635, miR-637, or miR-665 (Exiqon) for 2 h at 55 °C. The slides were washed in 0.2× SSC for 1 h at 60 °C and blocked for 1 h with 10 % fetal calf serum (FCS) in B1 (0.1 M Tris pH 7.5/0.15 M NaCl). Subsequently, slides were incubated with anti-digoxigenin-AP Fab fragments (1:2,500, Roche Diagnostics) in 10 % FCS in B1 overnight at 4 °C. The slides were reacted with 5-bromo-4-chloro-3-indolyl phosphate (BCIP) and nitroblue tetrazolium (NBT) substrates (NBT/BCIP stock solution, Roche Diagnostics) in B3 (0.1 M Tris pH 9.5/0.1 M NaCl/50 mM MgCl_2_) for 6–20 h at RT. Staining was terminated by washing the slides in PBS. Slides were mounted in 90 % glycerol in PBS or further processed for immunohistochemistry. Sections stained in parallel with scrambled LNA-DIG probe, a commonly used control for miRNA-ISH [30]), were devoid of staining.

### Immunofluorescent double labeling

For double labeling, in situ hybridization was followed by immunohistochemistry. Briefly, slides were blocked for 1 h at RT in 1 % FCS in PBS/0.2 % TritonX100 before primary antibody was applied overnight at 4 °C. Anti-glial fibrillary acidic protein (GFAP) antibodies (1:6,000, Dako Cytomation, Glostrup, Denmark) were used to localize the astrocytic marker GFAP. Donkey-anti-rabbit Alexa 488 (Invitrogen, Molecular Probes, Oregon, USA) was used as secondary antibody. Sections incubated without primary antibody were devoid of signal. Finally, all slides were reacted with 4′,6′-diamidino-2-phenylindole (DAPI) to fluorescently stain the nucleus. Images were taken using Axioscop 1 and Axiovert 2 microscopes (Carl Zeiss). Some images were pseudocolored using Photoshop CS2 (Adobe).

### Target analysis of deregulated miRNAs

To identify mRNAs that are targeted by miRNAs deregulated in mTLE, a set of genes involved in glutamatergic and GABAergic transmission, immune response and K^+^/water homeostasis was selected and the validated and predicted miRNA interactions of these genes were extracted from miRecords. The predictions were generated based on a minimal overlap of four prediction algorithms. The overlap between each miRNA interaction list and the 15 most up- and 15 most down-regulated miRNAs was determined by Venn analysis using a Web-based tool (http://www.pangloss.com/seidel/Protocols/venn.cgi).

### Bead-based ELISA

Bead-based ELISA was performed to determine CCL3, CCL22, and ICAM1 protein levels [31, 32]. In brief, 20 mg of hippocampal tissue from the same ten patients in each of the three patient groups used for miRNA profiling was homogenized in lysis buffer (Lysis M, Roche), sonicated, centrifuged at 4,500 × *g*, filtered through a 0.22-μm column and frozen at 0.5 μl/μl. Bead-bound and capture antibodies for CCL3 (capture: mouse monoclonal, detection: goat polyclonal), CCL22 (capture: mouse monoclonal, detection: chicken polyclonal) and ICAM1 (capture: mouse monoclonal, detection: sheep polyclonal) were used in a 50-μl homogenate. All antibodies were purchased from R&D systems (Abington, United Kingdom).

### Luciferase reporter assays

The psiCHECK™-2 vector (Promega) was used as a reporter for testing the ability of individual miRNAs to inhibit protein expression. This vector contains the coding sequences of both firefly and Renilla luciferases. While the firefly luciferase gene is constitutively transcribed and used for normalization, the Renilla gene contains a multiple cloning site (MCS) in its 3′untranslated region (UTR) enabling the introduction of miRNA-binding sites. miRNA-binding sites predicted by at least three different algorithms in miRecords were cloned into the psiCHECK™-2 vector. Oligonucleotides representing different predicted miRNA-binding sites (Table 2) were phosphorylated, annealed and cloned into the NotI and XhoI sites of the MCS. For reporter assays, HEK293 cells were seeded in 24-well plates (8 × 10^4^ cells/well) and transfected with 250 ng reporter construct and 25 pmol miRIDIAN miRNA or non-targeting control mimic (cel-miR-67; both from Dharmacon) per well using Lipofectamine 2000 (Invitrogen). Cells were harvested 24 h after transfection for analysis of luciferase activity using the Dual-Luciferase Assay System (Promega) and a Wallac Victor Luminometer. Relative luciferase activities were obtained by normalizing Renilla luciferase activity to that of firefly luciferase.

**Table 2:** Target Oligonucleotides for microRNA-binding sites

	Forward	Reverse
ICAM1		
miR-635	tcgagGAGTGCCCAGGGAATATGCCCAAGCTAgc	ggccgcTAGCTTGGGCATATTCCCTGGGCACTCc
miR-637_1	tcgagCATTGGCCAACCTGCCTTTCCCCAGAAGgc	ggccgcCTTCTGGGGAAAGGCAGGTTGGCCAATGc
miR-637_2	tcgagGGTCTCTGGCCTCACGGAGCTCCCAGTCCTgc	ggccgcAGGACTGGGAGCTCCGTGAGGCCAGAGACCc
miR-221&222	tcgagGAAGTGGCCCTCCATAGACATGTGTAGCATCAAAACgc	ggccgcGTTTTGATGCTACACATGTCTATGGAGGGCCACTTCc
CCL22		
miR-625	tcgagTGGGATTTGGGGGTTTTCTCCCCCAgc	ggccgcTGGGGGAGAAAACCCCCAAATCCCAc
miR-620	tcgagAACTCTCTGCATTCCCTGATCTCCATCCgc	ggccgcGGATGGAGATCAGGGAATGCAGAGAGTTc
miR-665	tcgagAGGCTGGTCTCAAACTCCTGGGCTCAAGCGATCCTCCTGGCTCgc	ggccgcGAGCCAGGAGGATCGCTTGAGCCCAGGAGTTTGAGACCAGCCTc
miR-635	tcgagCAAGGCATTGCTCGCCCAAGCAGgc	ggccgcCTGCTTGGGCGAGCAATGCCTTGc
CCL3		
miR-622	tcgagTGGCACCAAAGCCACCAGACTGACAgc	ggccgcTGTCAGTCTGGTGGCTTTGGTGCCAc

Table shows primers used to clone predicted miRNA-binding sites into the psiCheck-2 vector. Sequences are derived from the 3′UTR of *Ccl3*, *Ccl22* and *Icam1* in addition to NotI and XhoI restriction sites (indicated in *lower case*). The strongest miRNA-binding sites predicted by at least three different algorithms were tested

### Western-blot analysis

HeLa cells were transfected with 125 pmol miRIDIAN miRNA or non-targeting control mimic per well of a six-well plate using Lipofectamine 2000 (Invitrogen). At 48 h after transfection, cells were washed in ice-cold 1× PBS and lysed in 150 μl of lysis buffer (20 mM Tris pH 8, 150 mM KCl, 1 % Triton) supplemented with protease inhibitors (Complete; Roche) per well. Cell lysates were separated on a gradient NuPAGE (7–12 %) polyacrylamide gel (Invitrogen) and transferred to nitrocellulose blots. Blots were blocked overnight in 5 % non-fat milk powder in TBS-Tween. Next, the blots were incubated with primary antibodies in TBS-Tween for 1 h at RT [(mouse anti-α-tubulin (T-5168, 1:5,000, Sigma-Aldrich) and mouse anti-ICAM1 (sc-8439, 1:1,000, Santa Cruz)]. Following secondary antibody incubation, antibody binding was visualized using a chemiluminescence reagent kit (SuperSignal West Dura; Thermo Scientific) and quantified using ImageJ software. For each lane, the intensity of the α-tubulin band was used to normalize the ICAM1 signal. All relative ICAM1 intensities were compared to that of control transfected cells.

## Results

### Genome-wide microRNA expression profiling in human mTLE

We determined genome-wide miRNA expression profiles in hippocampal tissue from mTLE patients without (mTLE−HS) or with hippocampal sclerosis (mTLE + HS), and in autopsy control patients (Table 1), an experimental design which has been used previously [33–36]. The miRNA expression data were subjected to unbiased clustering by samples and miRNAs, and 130 miRNAs passed the filtering criteria on variation across samples (Online Resource 2a). A principle component analysis (PCA) of these miRNAs showed that miRNA expression patterns of individuals within each patient group were similar, but that profiles between groups differed. One mTLE + HS patient (Table 1; number 22) appeared to cluster with the mTLE−HS (W0) group. Indeed, neuropathological reassessment of this patient revealed only mild hippocampal sclerosis (W2 diagnosis).

**Fig. 1:**
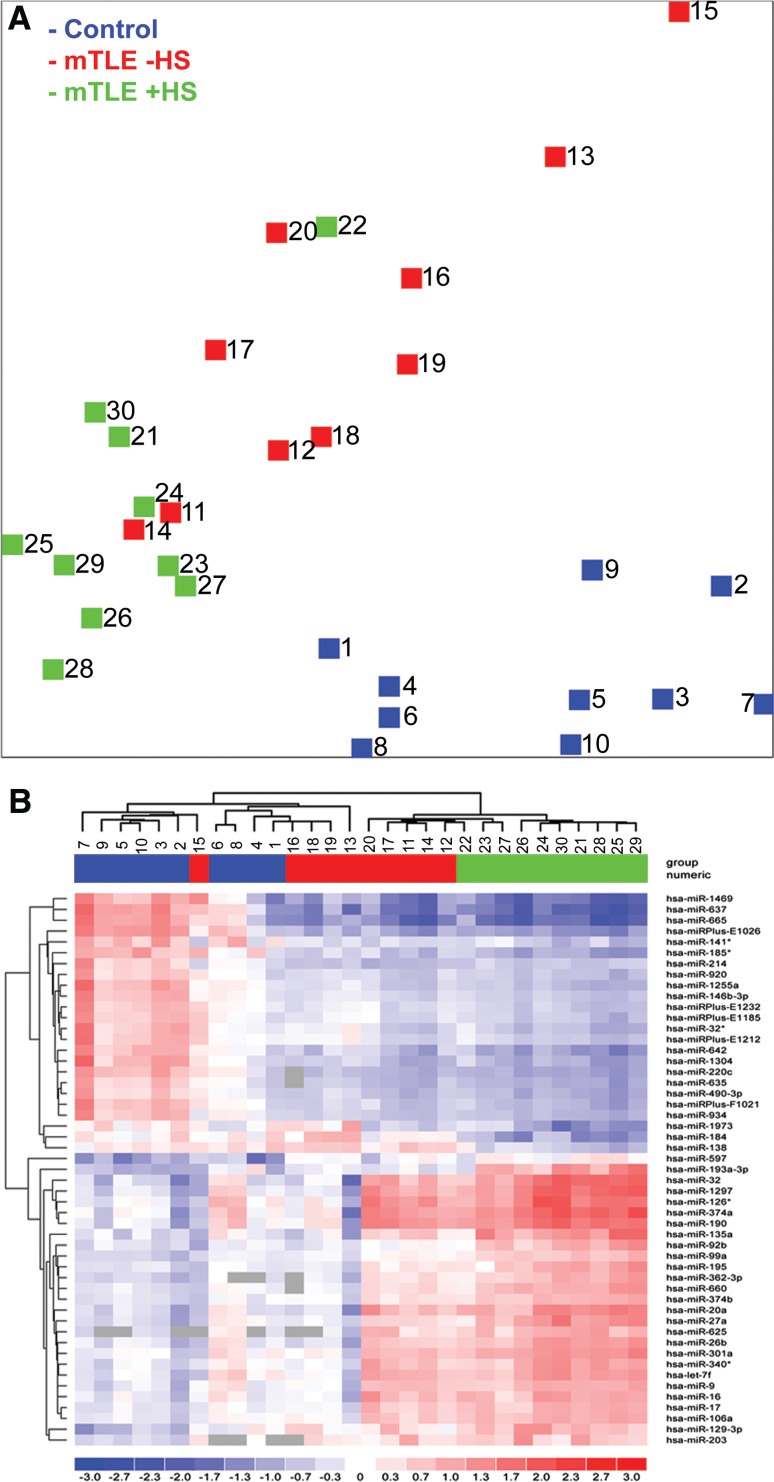
miRNA expression profiling in human mTLE. miRNA expression profiles were determined in hippocampal tissue of autopsy control (*blue*) and mTLE patients without (−HS; *red*) and with (+HS; green) hippocampal sclerosis using LNA-based microarray technology. **a** Principle component analysis (PCA) of the microarray data. Clustering of the samples using 130 miRNAs with the highest degree of variance (Table S1A: Sheet Exp. matrix (unsupervised), labeled in *green*). The PCA shows that individuals within a patient group cluster together, whereas the three groups segregate. **b** A heat map of 51 statistically significant miRNAs with a fold change of >2.0 depicted as a two-way hierarchical cluster (Table S1B: Sheet Exp. matrix (TTEST), labeled in *green*). *Blue* denotes down-regulated expression and *red* up-regulated expression compared to the mean. *Gray boxes* indicate probes without signal. Numbers 1–30 in **a** and **b** depict individual patients (Table 1)

**Fig. 2:**
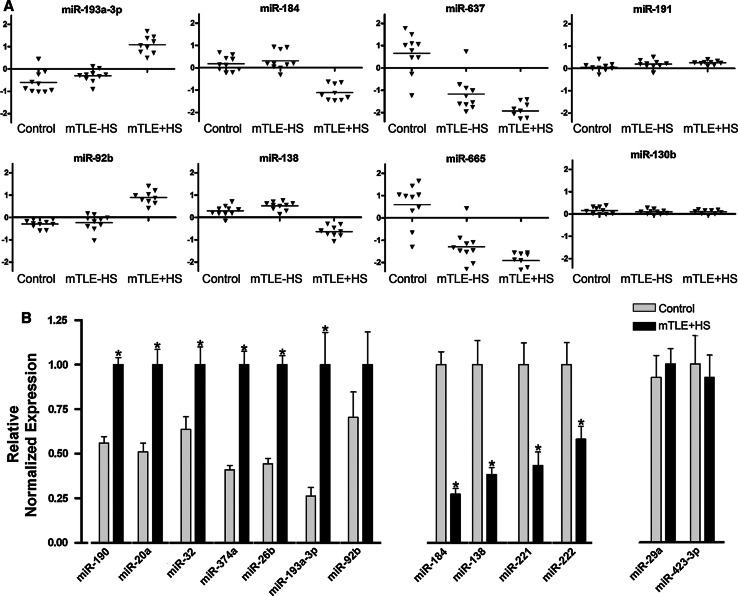
Differential expression of miRNAs in human mTLE. **a** Scatter plots of log^2^ Hy3/Hy5 ratios of representative miRNAs showing different expression profiles. *Triangles* represent individual patients. *Gray horizontal bars* indicate group means. miRNAs-193a-3p and 92b are up-regulated in mTLE + HS, and miR-184 and miR-138 are down-regulated in mTLE + HS. miR-637 and miR-665 are down-regulated in both mTLE patient groups. Several miRNAs including miR-191 and miR-130b do not show differential regulation between the three patient groups. **b** Validation of the microarray data by quantitative PCR (qPCR) on 11 candidate miRNAs in pooled patient samples (autopsy control and mTLE + HS). *Graphs* represent relative normalized expression with SEM. miR-29a and miR-423-3p served as normalization miRNAs. Significant change *p < 0.05

Analysis of changes in the expression of individual miRNAs identified 165 miRNAs with *p* values lower than 3.92 × 10^−05^ (Bonferroni corrected). Fifty-one of these miRNAs showed a fold change of >2.0 (Table 3) and were used in a two-way hierarchical clustering (Fig. 1b). This analysis revealed different patterns of expression (Fig. 2a). One set of miRNAs showed increased expression in the mTLE + HS group as compared to control and mTLE−HS (e.g., miR-193a-3p, miR-92b). Another set contained miRNAs with decreased expression in the TLE + HS group only (e.g., miR-184, miR-138). Interestingly, miR-221 and miR-222, which are derived from a common polycistronic precursor, were also down-regulated in mTLE + HS patients (fold change = 1.9). As expected, linear regression analysis revealed strong co-regulation of these two clustered miRNAs in our data (*R* ^2^ = 0.976), supporting the validity of the microarray. A third group contained miRNAs with decreased expression in both mTLE groups (e.g., miR-637, miR-665, miR-642). Analysis of miRNAs displaying significant but less prominent fold changes revealed additional patterns of expression changes, e.g., up- or down-regulated expression in mTLE−HS patients as compared to control and mTLE + HS (e.g., miR-890). Furthermore, the expression of a large set of miRNAs was similar in all three patient groups (e.g., miR-191 and miR-130b) (Fig. 2a).

**Table 3:** Strongly regulated microRNAs in mTLE

miRNA	mTLE−HS vs. control (ΔLMR)	mTLE + HS vs. control (ΔLMR)
let-7f	0.33	1.05
miR-9	0.52	1.06
miR-16	0.47	1.1
miR-17	0.44	1.07
miR-20a	0.45	1.51
miR-26b	0.21	1.18
miR-27a	0.34	1.34
miR-32	0.51	1.88
miR-32*	−0.7	−1.17
miR-92b	0.06	1.1
miR-99a	0.34	1.03
miR-106a	0.43	1.02
miR-126*	0.45	1.45
miR-129-3p	0.79	1.35
miR-135a	0.2	1.4
miR-138	0.22	−0.85
miR-141*	−1.05	−1.22
miR-146b-3p	−0.88	−1.16
miR-184	0.14	−1.24
miR-185*	−0.97	−1.49
miR-190	0.65	1.62
miR-193a-3p	0.3	1.53
miR-195	0.21	1.08
miR-203	0.81	1.06
miR-214	−1.08	−0.96
miR-220c	−1.16	−1.49
miR-301a	0.29	1.13
miR-340*	0.43	1.09
miR-362-3p	0.44	1.23
miR-374a	0.44	1.66
miR-374b	0.3	1.01
miR-490-3p	−0.81	−1.16
miR-597	0.94	1.14
miR-625	0.46	1.02
miR-635	−0.93	−1.22
miR-637	−1.83	−2.53
miR-642	−1.03	−1.54
miR-660	0.28	1.09
miR-665	−1.87	−2.38
miR-920	−0.73	−1
miR-934	−1.05	−1.27
miRPlus-F1021	−0.98	−1.31
miRPlus-E1026	−1.62	−1.96
miRPlus-E1185	−0.61	−1.05
miRPlus-E1212	−0.68	−1.08
miRPlus-E1232	−0.69	−1.09
miR-1255a	−1.04	−1.37
miR-1297	0.4	1.73
miR-1304	−1.13	−1.43
miR-1469	−1.32	−2.05
miR-1973	−0.2	−1.09

Those miRNAs are listed, which were at least two-fold and significantly regulated (*p* < 3.92 × 10^−05^) in mTLE−HS and/or mTLE + HS in comparison to control

Δ*LMR* difference in means of the log^2^ median ratios

To confirm the expression changes in the microarray data, 11 miRNAs were assayed by qPCR. Candidates showed significant differences between autopsy control and mTLE + HS patients and a fold change of >2 (miR-221/222 were selected because of their genomic organization and hippocampal enrichment). QPCR reactions (excluding patient #22 from the mTLE + HS group) for the selected miRNAs yielded expression changes to those obtained by microarray analysis (Fig. 2b).

### Differential miRNA expression in human mTLE

**Fig. 3:**
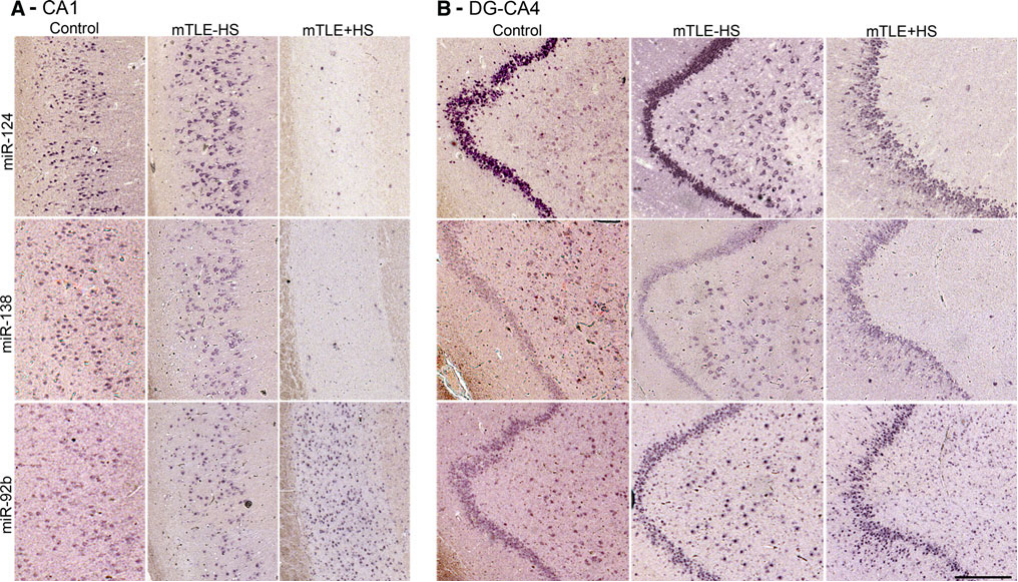
Changes in miRNA distribution in human mTLE. The spatial distribution of selected miRNAs across the three patient groups was determined by miRNA-ISH on consecutive hippocampal paraffin sections. Expression patterns (**a** CA1 subfield; **b** DG and CA4) are shown for three miRNAs in one subject per patient group (autopsy control, mTLE−HS and mTLE + HS). miR-138 and miR-92b were selected as examples of distinct expression pattern changes (up- and down-regulation). miR-124 served as a neuronal marker and technical control. Expression of miR-124 and miR-138 in neurons is almost completely lost in the CA1 and CA4 regions in mTLE + HS patients. In contrast, miR-92b expression is increased in small-sized cells in both CA1 and CA4 in mTLE + HS. Also note the granule cell dispersion that is characteristic of mTLE + HS in **b**. 1–4, cornu ammonis (*CA*) subfields 1–4; *DG*, dentate gyrus. *Scale bar* 200 μm

The specific distribution of several regulated miRNAs (identified in the microarray) in the control human hippocampus (Online Resource 1) prompted us to examine which cells contribute to the mTLE-associated changes in miRNA levels. Using miRNA-ISH, we compared the hippocampal localization of several strongly regulated miRNAs between all patient groups. Changes in hippocampal miRNA expression in autopsy controls, mTLE−HS and mTLE + HS patients are documented in Fig. 3 for two representative miRNAs [miR-138 (down-regulation) and miR-92b (up-regulation)]. The first change observed was a reduction in miRNA expression in the CA1, CA3, and CA4 subfields (observed for miR-124, miR-92b, miR-138) (Fig. 3a, b) in mTLE + HS patients. Moreover, in mTLE + HS patients expression of the selected miRNAs in the DG was diffuse as a result of granule cell dispersion (Fig. 3b).

**Fig. 4:**
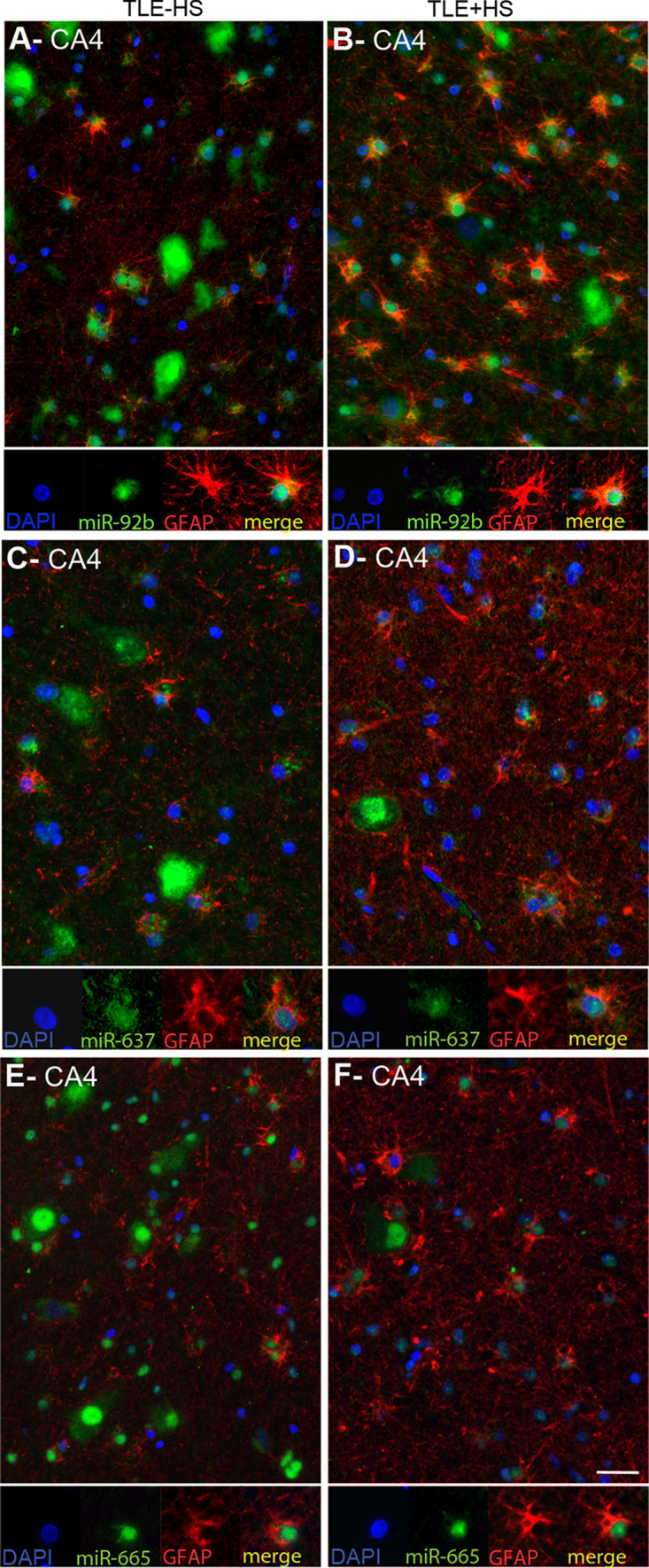
Expression of microRNAs in GFAP-positive astrocytes in mTLE. To characterize the small-sized cells expressing miR-92b, miR-637 and miR-665 in the hippocampus of mTLE patients, miRNA-ISH was combined with immunohistochemistry for glial fibrillary acidic protein (GFAP) on hippocampal sections of mTLE−HS and mTLE + HS patients. The miRNA-ISH signal is pseudocolored in *green*, nuclear DAPI staining in *blue* and GFAP labeling in *red*. The lower part of each panel shows images of individual astrocytes at a higher magnification. In mTLE, but not control (not shown), patients miR-92b (**a**, **b**), miR-637 (**c**, **d**) and miR-665 (**e**, **f**) expression is predominantly localized to the nucleus of GFAP-positive astrocytes. *Scale bar* 40 μm

Another prominent expression change was an increase in miRNA expression in small-sized cells in gliotic regions of the mTLE + HS hippocampus (CA1 and CA4; observed for miR-92b, miR-637, miR-665) (Fig. 3). To characterize these cells, miRNA-ISH was combined with immunohistochemistry for GFAP, an astrocyte marker. As expected, many of the small-sized cells expressing miR-92b, miR-637, or miR-665 in the CA1 and CA4 regions of mTLE + HS, and also mTLE−HS, patients were GFAP-positive (Fig. 4). In addition, small-sized GFAP-negative cells, possibly representing microglia, expressed the miRNAs.

### Abnormal nuclear distribution of miRNAs in neurons and astrocytes in mTLE

**Fig. 5:**
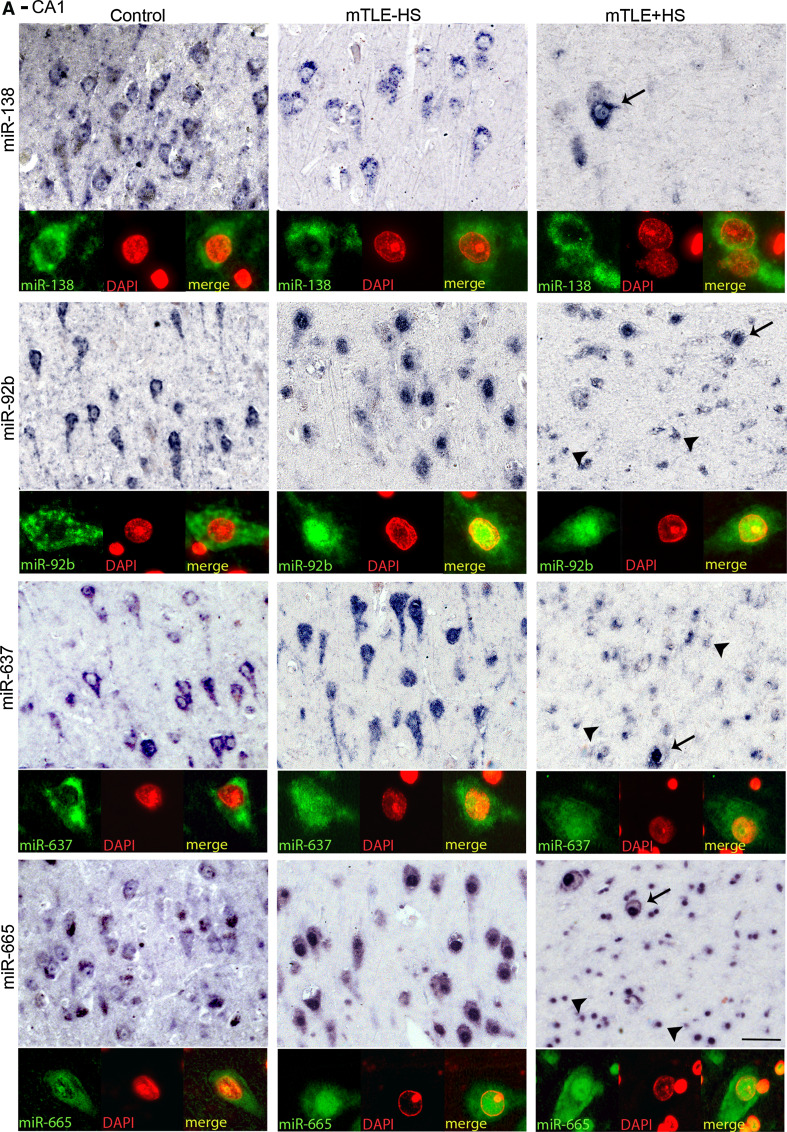

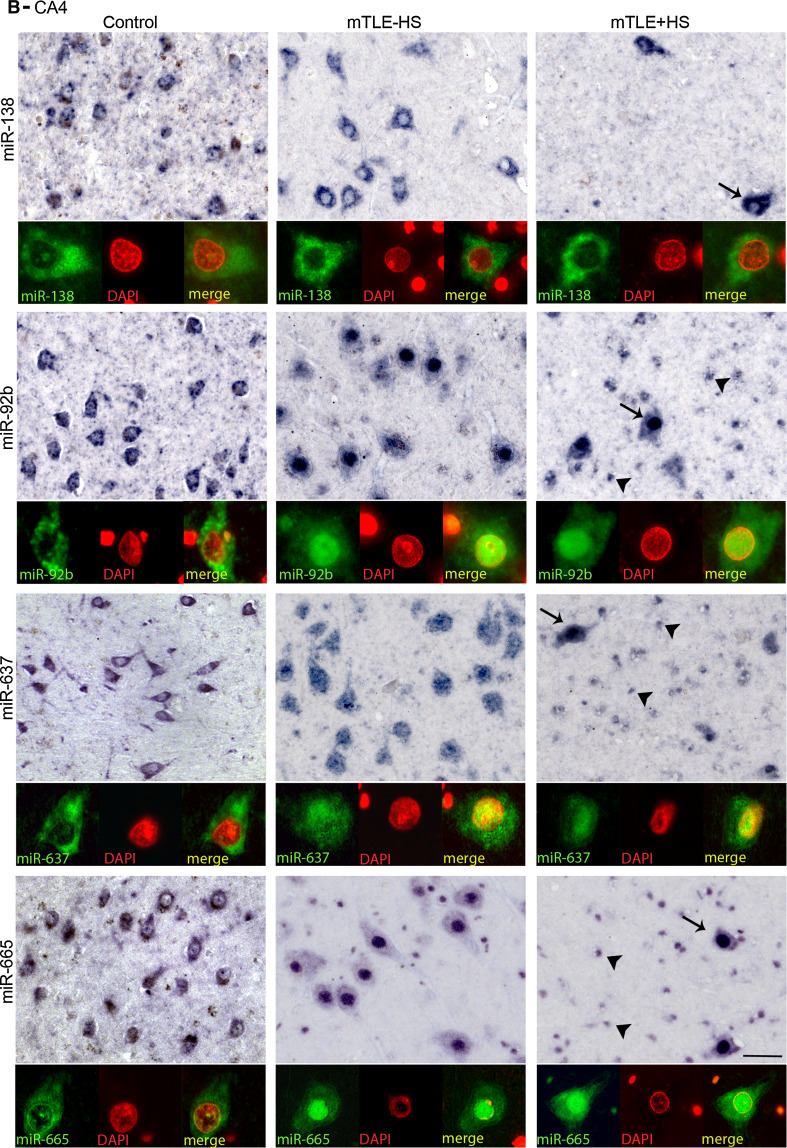
mTLE-associated nuclear mislocalization of miRNAs. Analysis of the miRNA-ISH data (as shown in Fig. 4 and 5) reveals a nuclear mislocalization for miR-92b, miR637, and miR665 in mTLE, but not for miR-138. In both CA1 (**a**) and CA4 (**b**), neuronal miRNA-138 expression is cytosolic in all three patient groups, a pattern observed for most miRNAs. In contrast, miR-92b, miR-637 and miR-665 are also found in the nucleus in mTLE−HS and mTLE + HS but not control patients. Double labeling with DAPI confirms this mTLE-associated nuclear localization, which is observed both in neurons (*arrows*) and small-sized cells (*arrowheads*). *Scale bar* 40 μm

Most miRNAs localize and function in the cytoplasm. Analysis of miRNA expression patterns in autopsy controls confirmed localization of miR-92b, miR-637, and miR-665 in the cytoplasm of neurons (Fig. 5a, b). In striking contrast, in mTLE patients their expression was prominent in the nucleus (shown by the co-localization with nuclear DAPI signals) in addition to cytoplasmic labeling. Nuclear signals for miR-92b, miR-637, and miR-665 were also observed in GFAP-positive astrocytes in mTLE patients (Fig. 4a–c). The atypical nuclear staining was observed in all mTLE patients, but in none of the autopsy controls. In contrast, the signal for miR-138 and for several other miRNAs (not shown) was confined to the cytoplasm in mTLE patients (Fig. 5a, b).

### The immune response as a target of miRNAs in mTLE

A single miRNA can regulate multiple different mRNAs and a single mRNA can be regulated by several miRNAs. Therefore, the mTLE-associated changes in miRNA expression and subcellular distribution observed in this study may have profound effects on biological functions. To study the biological significance of the most robust miRNA expression, we focused on genes and processes with firmly established roles in mTLE, i.e., glutamatergic/GABAergic transmission, the immune response, and glial K^+^ and water homeostasis (Table 4) [5, 37, 38]. The predicted miRNA interactions of the selected genes were extracted from miRecords (Online Resource 3) and compared to the 30 most regulated miRNAs (15 up- and 15 down-regulated; Online Resource 2). Many of the selected genes contained predicted binding sites for miRNAs regulated in mTLE. For several genes, more than 10 % of the total set of miRNAs predicted to target the gene were deregulated in mTLE. The most prominently targeted mRNAs were found in the immune response group. For example, 50, 31, and 14 % of miRNAs predicted to target CCL3, ICAM1 and CCL22, respectively, were deregulated in mTLE (Table 4). This is intriguing as immune cells and their inflammatory mediators play an important role in the pathophysiology of seizures and epilepsy [38].

**Table 4:** Deregulated miRNA target components of key pathways in mTLE

Protein name	Gene name	No predicted miRNAs	mTLE-regulated miRNAs	
			No	Name
Glutamate transmission				
GLUA1	*GRIA1*	7	0	
GLUA2	*GRIA2*	48	1	miR-203,
GLUA3	*GRIA3*	47	3	miR-32, miR-92b, miR-203
GLUA4	*GRIA4*	65	3	miR-26b, miR-27a, miR-625
NR1	*GRIN1*	31	3	miR-16, miR-195, miR-214
NR2A	*GRIN2A*	22	1	miR-597
NR2B	*GRIN2B*	16	2	miR-625, miR-642
NR2C	*GRIN2C*	1	0	
NR2D	*GRIN2D*	1	0	
mGLUR1	*GRM1*	46	4	miR-139-5p, miR-490-3p, miR-497, miR-642
mGLUR5	*GRM5*	21	0	
VGLUT1	*SLC17A7*	25	5	miR-17, miR-20a, miR-138, miR-620, miR-622
EAAT1	*SLC1A3*	31	2	miR-490-3p, miR-625
EAAT2	*SLC1A2*	167	17	miR-16, miR-17, miR-20a, miR-27a, miR-139-5p, miR-195, miR-203, miR-214, miR-221, miR-497, miR-620, miR-625, miR-635, miR-642, miR-660, miR-665, miR-934
EAAT3	*SLC1A1*	36	5	miR-9, miR-26b, miR-374a, miR-374b, miR-620
GS	*GLUL*	36	1	miR-625
PAG	*GLS*	37	1	miR-660
GABA transmission				
GABA_A_R α1	*GABRA1*	48	7	miR-16, miR-129-3p, miR-139-5p, miR-195, miR-203, miR-221, miR-222
GABA_A_R α2	*GABRA2*	5	0	
GABA_A_R α3	*GABRA3*	0	0	
GABA_A_R α4	*GABRA4*	121	6	miR-26b, miR-203, miR-374b, miR-620, miR-642, miR-660
GABA_A_R α5	*GABRA5*	9	1	miR-203
GABA_A_R α6	*GABRA6*	2	0	
GABA_A_R β1	*GABRB1*	6	0	
GABA_A_R β2	*GABRB2*	94	4	miR-9, miR-190, miR-203, miR-637
GABA_A_R β3	*GABRB3*	89	5	miR-27a, miR-203, miR-597, miR-622, miR-642
GABA_A_R γ1	*GABRG1*	80	6	miR-17, miR-26b, miR-135a, miR-221, miR-222, miR-597
GABA_A_R γ2	*GABRG2*	32	2	miR-203, miR-221
GABA_A_R γ3	*GABRG3*	2	0	
GABA_B_R 1	*GABBR1*	1	1	miR-620
GABA_B_R 2	*GABBR2*	53	7	let-7f, miR-9, miR-17, miR-20a, miR-106a, miR-139-5p, miR-203
Immune response				
IL-1α	*IL1A*	24	1	miR-146b-3p
IL-1β	*IL1B*	3	0	
IL-1Ra	*IL1RN*	28	0	
IL-5	*IL5*	7	1	miR-642
IL-6	*IL6*	15	1	let-7f
IL-7	*IL7*	16	1	miR-203
IL-10	*IL10*	26	3	let-7f, miR-27a, miR-597
IFN-α	*IFNA1*	1	0	
TNF-α	*TNF*	4	0	
CCL2	*CCL2*	8	0	
CCL3	*CCL3*	2	1	miR-622
CCL4	*CCL4*	3	0	
CCL5	*CCL5*	8	0	
CCL22	*CCL22*	36	5	miR-597, miR-620, miR-625, miR-635, miR-665
VEGF	*VEGFA*	73	5	*miR*-*16, miR*-*17, miR20a, miR106a*, miR-637,
ICAM1	*ICAM1*	16	5	miR-203, *miR*-*221, miR*-*222,* miR-635, miR-637,
K^+^ buffering				
AQP4	*AQP4*	93	4	miR-203, miR-597, miR-622, miR-635
KIR4.1	*KCNJ10*	52	6	miR-17, miR-20a, miR-106a, miR-298, miR-635, miR-637

miRNA target predictions were performed for groups of genes and pathways implicated in mTLE. The number of miRNAs predicted to interact with the mRNAs of the indicated genes is listed followed by the number and names of mTLE-deregulated miRNAs within this predicted mRNA-miRNA set. *Italic* miRNAs have validated interactions with the listed target transcript

*AQP4* aquaporin 4; *CCL* chemokine (C–C motif) ligand; *EAAT1–3* excitatory amino acid transporter 1–3; *GABA*_*A*_ *R α1–γ3* GABA-A receptor subunits α1–γ3; *GABA*_*B*_ *R 1–2* GABA-B receptor subunits 1–2; *GLUA1-4* glutamate receptor, ionotropic AMPA subunit 1–4; *GS* glutamine synthetase; *ICAM1* intercellular adhesion molecule 1; *IFN-α* Interferon-α; *IL* Interleukin; *Kir4.1* glial inwardly rectifying potassium channel Kir4.1; *mGluR* metabotropic glutamate receptor; *NR1–2b* NMDA subunit 1–2b; *PAG* phosphate activated glutaminase; *TNF-α* tumor necrosis factor; *VEGF* vascular endothelial growth factor; *vGLUT1* vesicular glutamate receptor 1

**Fig. 6:**
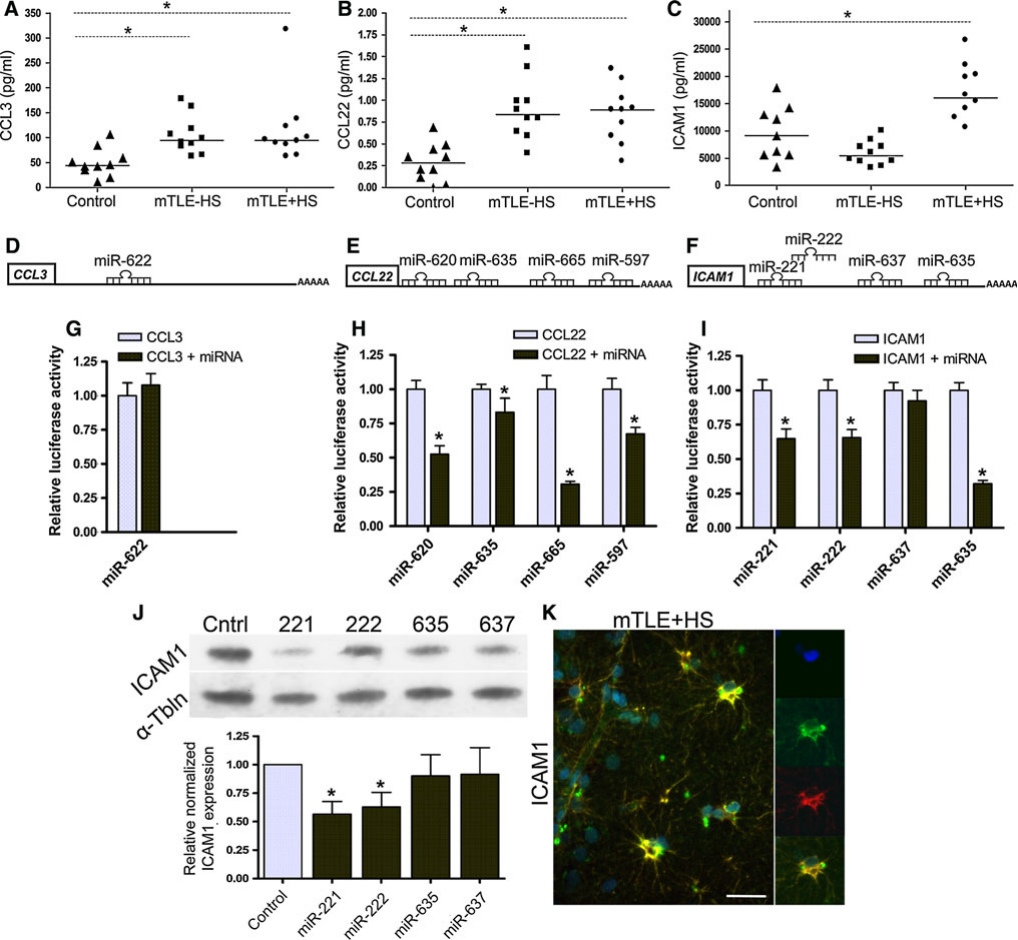
microRNAs deregulated in mTLE target the immune response. Quantitative ELISA measurements on the samples used for miRNA profiling (Table 1; *n* = 10 per group) show an increase in hippocampal expression of CCL3 (**a**) and CCL22 (**b**) in mTLE patient groups, and increased expression of ICAM1 (**c**) in mTLE + HS patients. Symbols represent individual patients, horizontal lines group means. 3′UTRs of CCL3 (**d**), CCL22 (**e**) and ICAM1 (**f**) with the relative location of the strongest predicted binding sites for the miRNAs that were most robustly regulated in mTLE. Luciferase activity in HEK293 cells transfected with the psiCheck-2 vector containing the binding sites indicated in **d**–**f** and corresponding miRNA mimic (*black bar*) or non-targeting control mimic (*light blue bar*). Levels of Renilla luciferase reporter activity were normalized to the levels of constitutively expressed firefly luciferase. The relative normalized means as compared to control ± SD (*n* = 3 independent experiments) are shown. Significant differences between the control miRNA and the miRNAs predicted to target CCL3, CCL22, and ICAM1 are indicated (*p* < 0.05). **j** Lysates from HeLa cells transfected with miRNA mimics for miR-221, 222, 635, or 637 or non-targeting control were subjected to Western blotting for ICAM1. Upper panel shows a representative blot incubated with antibodies against ICAM1 and α-tubulin (α-Tbln). Lower panel shows microdensitometry from four independent experiments. Normalized means ± SEM are shown. **k** Double immunofluorescent labeling on hippocampal tissue of a mTLE + HS patient reveals co-labeling of ICAM1 protein (*green*) with the astrocyte marker glial fibrillary acidic protein (GFAP) (*red*). Small panels on the *right* show images of an individual astrocyte at a higher magnification. Significant change *p < 0.05. *Scale bar* 40 μm

If miRNAs are important for modulating the immune response in mTLE, changes in miRNA expression are expected to induce reciprocal changes in the expression of immune proteins. Indeed, multiplex immunoassays (in the same patient samples used for miRNA profiling) revealed an up-regulation of CCL3 and CCL22 in both mTLE groups and a specific up-regulation of ICAM1 in mTLE + HS patients (Fig. 6a–c). Having identified (1) increased expression for CCL3, CCL22, and ICAM1, and (2) several down-regulated miRNAs that may target these cues in the same tissue (Table 4), we tested these miRNAs for their targeting of the 3′UTR of *CCL3*, *CCL22*, and *ICAM1*. One or more strong binding sites were examined for each miRNA in luciferase assays (Table 2), excluding miRNAs with inconsistent or low array signals (Fig. 6d–f). miR-622 did not target the 3′UTR of *CCL3* (Fig. 6g). In contrast, miR-597, miR-620, miR-625, miR-665 targeted *CCL2*. *ICAM1* was a target for miR-221, miR222, and miR-635 (Fig. 6h, i).

The potential regulation of ICAM1 by miRNAs is intriguing as ICAM1 protein, but not mRNA, levels are changed in human mTLE, hinting at changes at the post-transcriptional level (Fig. 6c) [3, 39]. Therefore, we tested the effect of miRNAs on endogenous ICAM1 expression. Transfection of miR-221 and miR-222 mimics into HeLa cells strongly suppressed endogenous ICAM1 expression (Fig. 6j). A smaller but non-significant effect was observed for miR-635 and miR637.

**Fig. 7:**
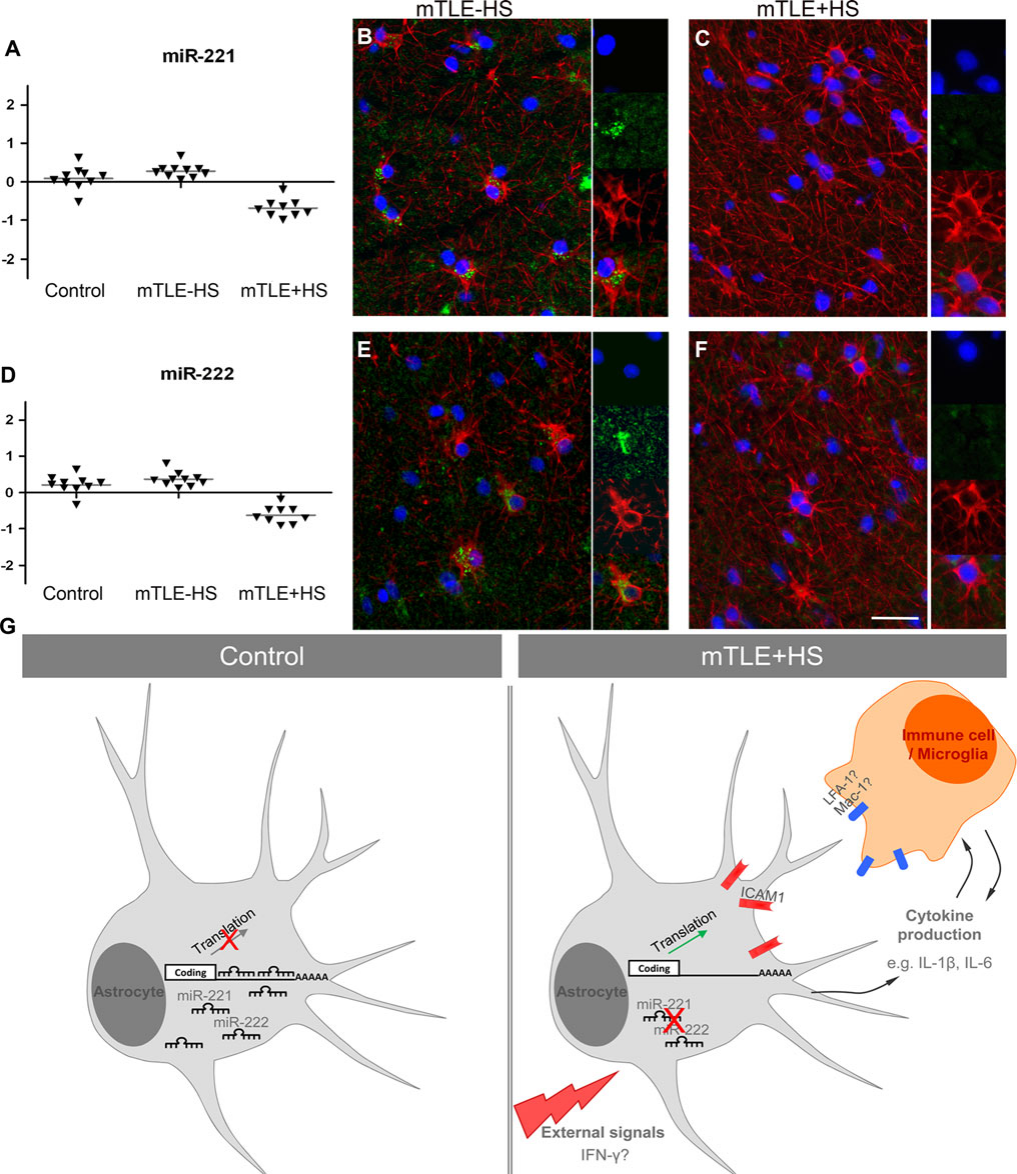
Decreased astrocyte-associated expression of miR-221 and miR-222 in mTLE + HS. **a**, **d** Scatter plots of log^2^ Hy3/Hy5 ratios for miR-221 and miR-222. *Triangles* represent individual patients and *gray horizontal bars* group means. **b**, **c**, **e**, **f** miRNA-ISH combined with immunofluorescent labeling for glial fibrillary acidic protein (GFAP). The miRNA-ISH signal is pseudocolored in *green*, nuclear DAPI staining in *blue* and GFAP labeling in *red*. The *right part* of each panel shows images of individual astrocytes at a higher magnification. miR-221 and miR-222 are detected in GFAP-positive astrocytes in controls (not shown) and mTLE−HS patients (**b**, **e**). In contrast, expression of miR-221 and miR-222 is absent or weak in astrocytes of the mTLE + HS hippocampus (**c**, **f**). **g** Hypothetical model of miRNA-regulated expression of ICAM1 in astrocytes in mTLE. In mTLE + HS, expression of miR-221 and miR-222 is down-regulated in astrocytes in the hippocampus as compared to control. These miRNAs target the 3′UTR of ICAM1 and reduce ICAM1 expression. Therefore, reduced miR-221 and miR-222 expression in mTLE + HS may induce enhanced astrocyte-associated expression of ICAM1. In line with this model, ICAM1 protein expression is increased in astrocytes in mTLE + HS patients. Astrocyte-associated ICAM1 has been associated with the recruitment, accumulation, and activation of leukocytes and microglia. These cells express ICAM1-binding partners such as LFA-1 and Mac-1 and ICAM1-LFA-1/Mac-1 interactions can trigger the production of inflammatory mediators by astrocytes and immune cells. These effects may contribute to the enhanced and sustained immune response observed in the mTLE + HS hippocampus. The signals that trigger changes in astrocyte-associated miRNA expression are unknown but may include factors such as IFN-γ, which can regulate ICAM1 expression at the post-transcriptional level. *Scale bar* 40 μm

Increased expression of ICAM1 is associated with different cell types in neural disorders including epilepsy [39–42]. Immunohistochemistry for ICAM1 in our mTLE patient material revealed enhanced ICAM1 expression in blood vessels and GFAP-positive astrocytes in the mTLE + HS hippocampus (Fig. 6k). This is reminiscent of recent observations in an experimental model of mTLE [42]. Microarray and qPCR experiments show that expression of miR-221 and miR-222 is down-regulated in mTLE + HS (Figs. 2b; 7a, b). In line with these data, we found that in mTLE + HS miR-221 and miR-222 expression was reduced in astrocytes in the CA1, CA3 and CA4 regions compared to control and mTLE−HS patients (Online Resource 1; Fig. 7b, c, e, f). No specific signals were detected in blood vessels. In all, these experiments show a reciprocal regulation of miR-221/222 and ICAM1 in astrocytes in mTLE + HS.

## Discussion

Discrepancies between mTLE-associated changes at the mRNA and protein level provide support to the idea that post-transcriptional regulation is affected in mTLE. For example, ICAM1 protein levels are up-regulated in the sclerotic mTLE hippocampus, but this change is not detected at the mRNA level (Fig. 6) [3, 39]. In the present study, analysis of miRNA signatures in mTLE patients revealed different levels of miRNA deregulation (changes in expression and subcellular distribution) and led us to identify astrocytes and the immune response as a target of deregulated miRNAs in mTLE. These findings extend the current concepts of mTLE pathogenesis to the level of miRNA-mediated post-transcriptional gene regulation. Because of the central role of astrocytes and the immune response, our results may have implications for other neurological and neurodegenerative disorders.

### microRNAs target components of the immune response in mTLE

We used a selection of genes/cellular processes associated with mTLE to begin to address the significance of our observations (Table 4). Among the selected candidates, inflammatory mediators were most prominently targeted by deregulated miRNAs. This is in line with the idea that inflammation may play a central role in epilepsy [38].

In vitro experiments revealed that miR-221 and miR-222 target the 3′UTR of ICAM1 (CD54). ICAM1 mediates interactions with other (immune) cells to influence processes such as inflammation [43]. In line with previous studies, we observed an increase in ICAM1 expression in blood vessels and GFAP + astrocytes in the mTLE + HS hippocampus [40, 42, 44]. Astrocytes contribute significantly to the pathogenic process of epilepsy [45]. Although the function of astrocyte-associated ICAM1 remains poorly understood, it has been proposed to mediate leukocyte accumulation, microglia recruitment, and cytokine production (e.g., IL-1β, IL-6) [40, 44, 46, 47].

In our study, miR-221 and miR-222 were expressed in astrocytes, but not in blood vessels, and their expression was reduced in mTLE + HS material. Interestingly, ICAM1 has been validated as a target for miR-221 or miR-222 in cholangiocytes, epithelial and cancer cells. In these cells, physiological stimuli induce different biological effects that depend on the miRNA-mediated regulation of ICAM1 expression [48–50]. Thus, miRNAs not merely fine-tune ICAM1 expression, but can robustly regulate ICAM1 expression to trigger biologically meaningful effects. Based on our findings and the current knowledge of astrocyte-associated ICAM1, we propose that the down-regulation of miR-221 and miR-222 in mTLE + HS is linked to a local up-regulation of ICAM1 in astrocytes. Interestingly, recent work has shown that miR-222 can regulate ICAM1 in glioma cells [49]. Enhanced ICAM1 expression may then contribute to the release of other inflammatory mediators and the recruitment of immune cells, thereby augmenting and/or sustaining the immune response (Fig. 7g).

Previous work indicates that the expression of ICAM1 is regulated post-transcriptionally in mTLE. Our results provide support to this idea by revealing a down-regulation of miRNAs that target ICAM1. The factors that trigger this decrease, and thereby potentially enhance ICAM1 levels, are unknown. ICAM1 protein expression can be regulated by a variety of signals [43]. Intriguingly, some of these signals, such as IFN-γ, have been shown to regulate ICAM1 at the post-transcriptional level [46].

Post-transcriptional regulation by miRNAs affects about a third of all protein-coding genes. Given the abundant and robust changes in miRNA expression reported in our study, one has to assume that in addition to CCL22 and ICAM1 the expression of many other proteins is regulated by miRNAs in mTLE. Indeed, our analysis of a limited number of genes and cellular processes (Table 4) revealed several interesting candidates for such regulation. Further studies are needed to establish the implications of these predicted interactions.

### Nuclear mislocalization of miRNAs in human mTLE

miRNAs are generally considered to be cytoplasm-localized regulatory RNAs. However, in our study a specific subset of miRNAs (miR-92b, miR-637, and miR-665) also displayed high levels of expression in the nucleus of neurons and glial cells in mTLE tissue but not controls. To the best of our knowledge, our study is the first to unveil a disease-associated nuclear mislocalization of miRNAs. Preliminary studies show a similar nuclear mislocalization of miR-92b in the pilocarpine-induced status epilepticus rat model of TLE (our own unpublished observations). It is therefore tempting to speculate that the nuclear mislocalization of miRNAs may be part of mTLE pathogenesis. It is unclear why these miRNAs aberrantly localize in the nucleus. One possibility is that they relocalize to the nucleus from the cytoplasm. Relocalization of miRNAs from the cytoplasm to the nucleus has been reported previously. For example, miR-29b contains a specific hexanucleotide sequence that directs its nuclear localization [51]. Furthermore, an active karyopherin-based shuttling system for cytoplasmic-nuclear transport of miRNAs has been reported. Final processing of miRNAs normally occurs in the cytoplasm and the LNA miRNA-ISH probes used in this study are thought to selectively detect fully processed, mature miRNAs, providing further support for a cytoplasm-to-nucleus shuttling model in mTLE. What could be the functional consequences of the nuclear mislocalization of miRNAs? Due to their nuclear localization miR-92b, miR-637, and miR-665 may fail to regulate their normal cytoplasmic target transcripts. In addition, they may acquire new functions in the nucleus such as transcriptional regulation. Both scenarios will have profound effects on protein expression. Future studies will need to focus on revealing the full set of miRNAs mislocalized in the nucleus in mTLE and on the causes and consequences of this abnormal distribution.

### Differential microRNA expression in mTLE: cause and/or consequence?

Several different patterns of miRNA expression changes were detected in mTLE patients. It is possible that some of these changes are seizure- and/or drug-induced or a consequence of the gliosis or neuron loss that characterize mTLE [52, 53]. Further detailed cellular analysis per miRNA will be required to investigate which changes are solely related to neuron loss and/or gliosis. Nevertheless, such differences in miRNA expression may still be relevant for the disease process, e.g., in the recurrence of seizures or the regulation of gliosis. It is also important to note that statistical analyses did not reveal any effect of confounding factors such as postmortem delay or age. Other types of miRNA expression changes, e.g., a specific down-regulation of astroglial miR-221 and miR-222 expression in mTLE + HS (Fig. 6) or mTLE−HS specific alterations, do not correlate directly with gross morphological changes, seizures or medication, the latter two of which are common to both mTLE patient groups (Table 1). These changes indicate that while some alterations in miRNA expression may be shared by both mTLE groups, others are unique to mTLE−HS or mTLE + HS. This is also supported by the clearly segregated miRNA signatures of the different patient groups in the PCA plot (Fig. 1a). Since the human material used in this study represents an end-stage of the epileptogenic process, an important future goal is to determine whether the changes in miRNA expression or distribution represent a cause or a consequence of the disease process. A causal role for miRNAs in epilepsy is suggested by recent animal studies reporting changes in miRNA levels already during the process of epileptogenesis [18–21]. In the future, manipulation of the here identified deregulated miRNAs in animal models of TLE will help to address the functional, and potentially pathogenic, role of changes in the expression and subcellular distribution of these small RNAs.

### Electronic supplementary material

Below is the link to the electronic supplementary material.

Supplementary material 1 (DOCX 12 kb)

Supplementary material 2 (XLS 922 kb)

Supplementary material 3 (XLS 290 kb)

Supplementary material 4 (XLS 615 kb)

Supplementary material 5 (XLS 255 kb)

Supplementary material 6 (JPG 4.59 mb)
