# Supplementary material for: Genome-wide microRNA profiling of human temporal lobe epilepsy identifies modulators of the immune response
Source: Cell Mol Life Sci. 2012 Apr 26;69(18):3127–45. doi: 10.1007/s00018-012-0992-7 (PMC3428527; doi:10.1007/s00018-012-0992-7)
Supplement: Supplementary file 1 — Supplementary material 1 (DOCX 12 kb) [file 18_2012_992_MOESM1_ESM.docx]

**Supplementary Material**

**Online Resource 1. Spatial distribution of microRNAs in the human hippocampus.**

The normal spatial distribution of many of the miRNAs that are regulated in mTLE in our study is unknown. Therefore, LNA probe-based miRNA *in situ* hybridizations (miRNA-ISH) were performed for several of the most affected miRNAs on autopsy control tissue. Spatial expression pattern of miRNAs determined by miRNA-ISH on consecutive paraffin sections from hippocampal tissue of a representative control patient is shown. The expression patterns for each miRNA were consistent across patients. The neuronal miRNA miR-124 served as a technical control and neuronal marker. All selected miRNAs were detected in the hippocampus by miRNA-ISH albeit at different levels of expression. miR-124, miR-20a, miR-92b, miR-138, miR-222, miR-637 and miR-665 were robustly expressed in the dentate gyrus (DG) and cornu ammonis (CA) regions (1-4). Only low signals were detected for miR-193a-3p. miR-221 was predominantly expressed in the granular cell layer of the DG, whereas weaker signals were detected in the CA regions. Overall, these experiments validate the array data and reveal expression of miRNAs in different subregions of the human hippocampus. Scale bar, 1000µm.

**Online Resource 2**

a) Spreadsheet containing the expression matrices of all Hy3/Hy5 ratios across patients. b) PCA plot data labeled in green. c) Heatmap data depicted in green (Supplementary data for Fig. 1)

**Online Resource 3**

Spreadsheet containing detailed information on the reverse analysis approach (Table 4). For each gene details on process, protein name and gene ID are listed. In a separate worksheet, all predicted miRNA-gene interactions and those present in the regulated set are indicated.
