# Supplementary figures and images for: Genome-wide microRNA profiling of human temporal lobe epilepsy identifies modulators of the immune response
Source: Cell Mol Life Sci. 2012 Apr 26;69(18):3127–45. doi: 10.1007/s00018-012-0992-7 (PMC3428527; doi:10.1007/s00018-012-0992-7)

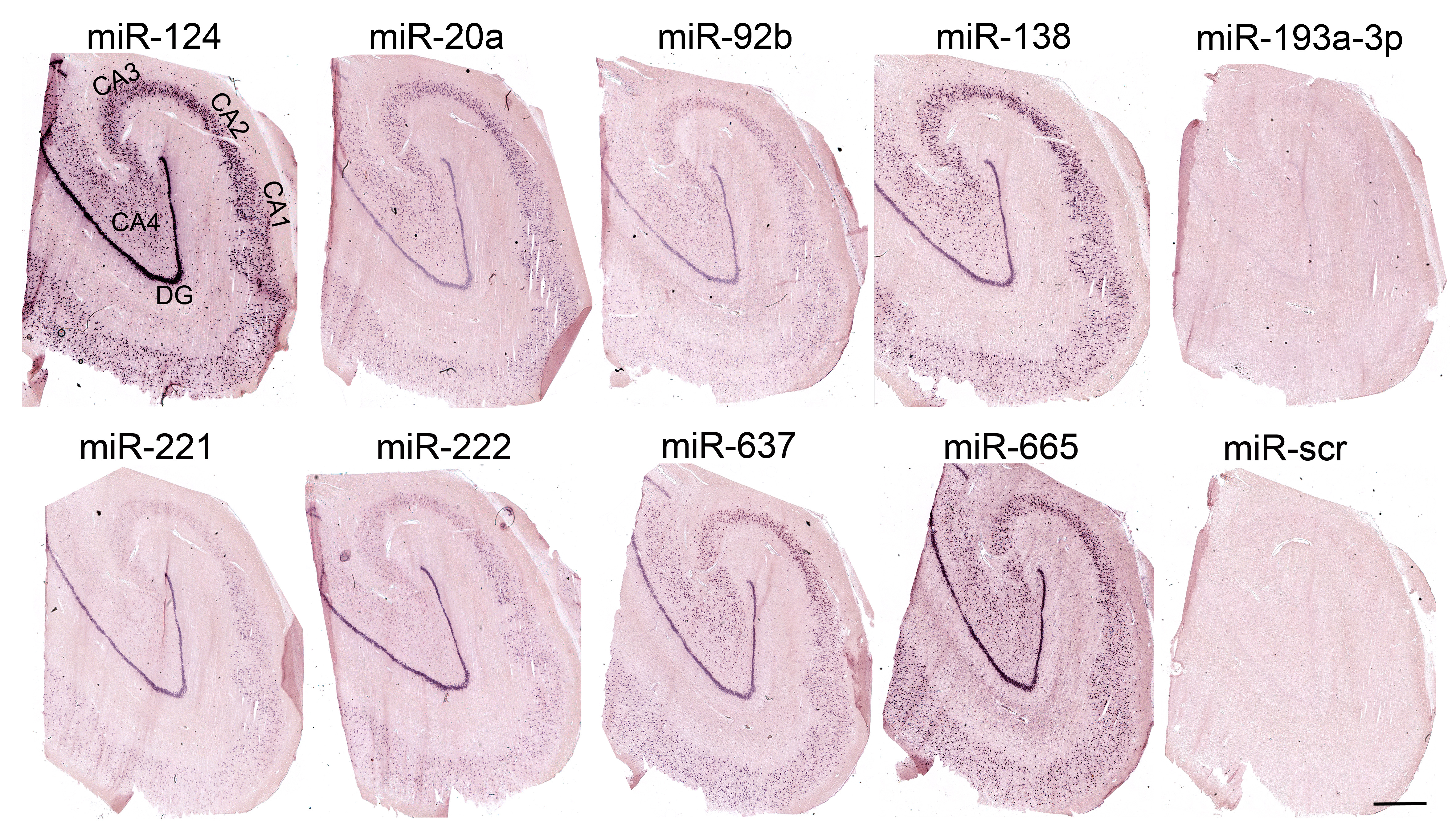

Supplement: Supplementary file 6 — Supplementary material 6 (JPG 4.59 mb) [file 18_2012_992_MOESM6_ESM.jpg]
